# Mucogingival Treatment of a Gingival Invagination After Tooth Extraction: A 3‐Year Follow‐Up After a Perio‐Ortho Multidisciplinary Approach

**DOI:** 10.1155/crid/5080226

**Published:** 2026-07-15

**Authors:** Jennifer Alberichi, Mariana Rojas, Juan Carlos Cometti, Andrea Pilloni

**Affiliations:** ^1^ Department of Comprehensive Rehabilitation of Medium and High Complexity, School of Dentistry, University of Buenos Aires, Buenos Aires, Argentina, uba.ar; ^2^ Section of Periodontics, Department of Oral and Maxillofacial Sciences, Sapienza University of Rome, Rome, Italy, uniroma1.it; ^3^ Department of Orthodontics and Maxillary Orthopedics, School of Dentistry, University of Buenos Aires, Buenos Aires, Argentina, uba.ar

**Keywords:** connective tissue graft, gingival cleft, mucogingival, orthodontics, periodontal plastic surgery, tooth extraction

## Abstract

**Background:**

Gingival invagination is a relatively common occurrence following orthodontic closure of extraction sites. The present paper reports a combined periodontal and orthodontic treatment in a patient after lower incisor tooth extraction, in which periodontal plastic surgery was performed prior to the orthodontic space closure.

**Case Report:**

A 28‐year‐old female patient was treated for orthodontic concerns. The comprehensive treatment plan encompassed periodontal supportive therapy, tooth extraction, and periodontal plastic surgery utilizing the “connective tissue platform technique.” Postsurgery, orthodontic treatment was administered with Invisalign aligners.

**Results:**

Radiographs and clinical examinations conducted 3 years after treatment completion confirm the long‐term stability of the outcomes, both in terms of function and esthetics.

**Conclusion:**

Gingival invagination after mandible incisor extraction could be successfully treated by an additional application of a periodontal plastic surgery technique, which, combined with a posterior orthodontic treatment, solved a multidisciplinary treatment challenge.

**Key Finding**

Gingival invagination after extraction could be successfully treated by an additional application of a periodontal plastic surgery technique.

**Key Points**

1. **What new information is this case providing?**

This case introduces a novel indication for periodontal plastic surgery to address gingival invagination postmandibular incisor extraction, utilizing the “connective tissue platform technique” by Zucchelli et al. for combined horizontal and vertical soft tissue augmentation.

2. **What are the keys to successful management of these cases?**

Interdisciplinary collaboration between periodontists and orthodontists, careful selection of surgical technique, and periodontal supportive therapy are essential for long‐term success.

3. **What are the limitations to success in this case?**

The lack of scientific evidence and well‐designed studies in this area highlights a limitation in evidence‐based treatment options for similar cases.

## 1. Introduction

Tooth extractions are a common necessity during orthodontic therapy, particularly in the management of tooth size‐arch length discrepancies [1]. As orthodontic space closure is executed, clinicians frequently encounter the emergence of gingival invaginations or clefts, presenting a clinical challenge that may contribute to orthodontic relapse and jeopardize periodontal health [2].

These gingival clefts (GCs) appear as linear invaginations of the interproximal gingival tissue and are known to affect a significant proportion of patients, with reported rates ranging from 35% to 100% [3–6]. However, the mechanisms and clinical conditions that favor their occurrence have yet to be clearly established.

Two current theories are aimed at elucidating the etiology of GCs. The first attributes their formation to changes in the underlying alveolar bone architecture of the extraction site, often occurring due to trauma, resorption, or other factors [2, 7, 8]. The second explanation centers on the transseptal fiber system; rather than being remodeled during tooth movement, these fibers are displaced, causing tissue bunching, pressure on the underlying bone, and ultimately the invagination of the gingival tissues [9, 10]. Although distinct, both mechanisms underlie the same clinical challenges associated with GCs: delayed or incomplete space closure, relapse, reduced interdental bone height, difficulty maintaining adequate plaque control, and impairment of the esthetic outcome [11].

In addressing this clinical challenge, the introduction of periodontal plastic surgery as a treatment option for GCs presents a potential therapeutic alternative in patient care and interdisciplinary collaboration between periodontists and orthodontists. The comprehensive management of these defects through surgical intervention holds the promise of enhancing patient outcomes and overall satisfaction. Moreover, these surgical principles are not exclusive to gingival invaginations. Pre‐existing recessions and soft tissue phenotype augmentation before orthodontic tooth movement represent valid indications, particularly when teeth are positioned—or planned to be moved—outside the alveolar bone envelope, where reduced cortical bone and gingival tissue thickness predisposes the site to recession. In these scenarios, addressing the gingival phenotype prior to orthodontic loading may serve both a therapeutic and a preventive purpose [12–14].

The aim of this paper is to present the long‐term outcomes following a mucogingival approach for a patient with a GC after tooth extraction, as part of a multidisciplinary (perio‐ortho) treatment.

## 2. Case Presentation

**Figure 1:**
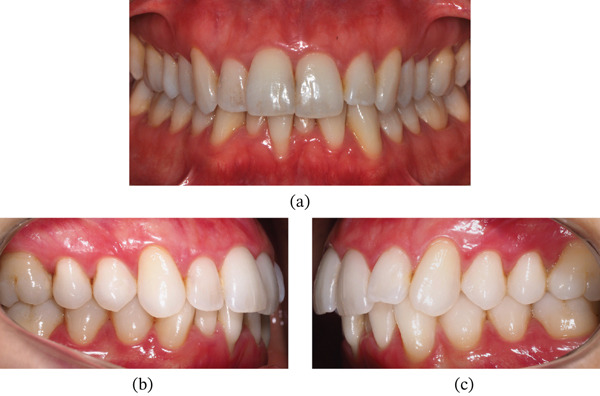
Baseline intraoral situation (a) frontal view, (b) right lateral view, and (c) left lateral view.

**Figure 2:**
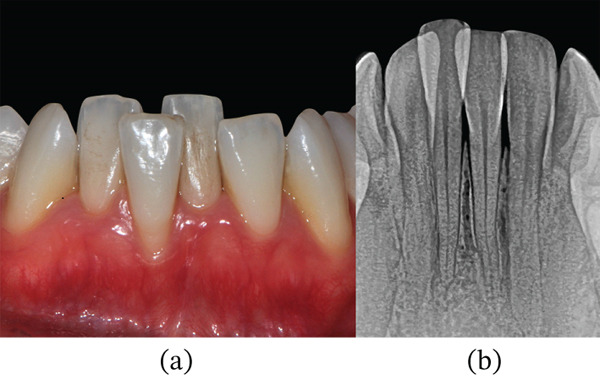
Baseline: (a) clinical and (b) radiographic view.

**Figure 3:**
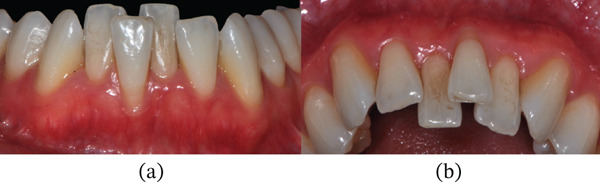
Baseline: (a) buccal and (b) occlusal view.

A 28‐year‐old female patient was referred to the University of Buenos Aires to start an orthodontic treatment. The patient′s medical history was unremarkable. A comprehensive clinical and radiographic evaluation was performed. From an orthodontic point of view, the patient presented with a Class I malocclusion (canine and molar Class I relationship), with a cusp‐fossa intermaxillary occlusal relationship (tooth to two teeth) on both sides of the dental arch, with Bolton discrepancy on upper lateral incisors and lower incisors crowding from right to left lower canines. Periodontally, the patient was healthy with a reduced periodontium and presented gingival recessions [15]. The treatment plan encompassed periodontal supportive therapy [16], lower incisor extraction, periodontal plastic surgery, and orthodontic treatment using aligners (Figures 1, 2, and 3).

**Figure 4:**
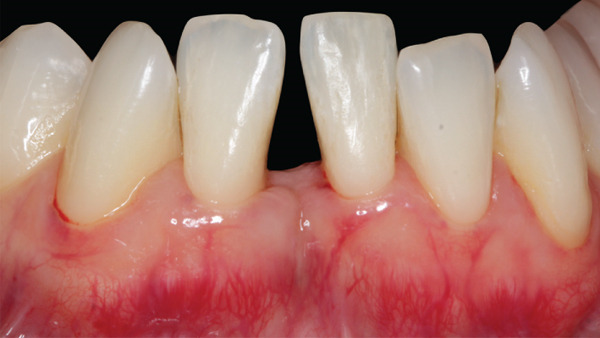
Presurgery, 45 days after tooth extraction. Buccal view.

**Figure 5:**
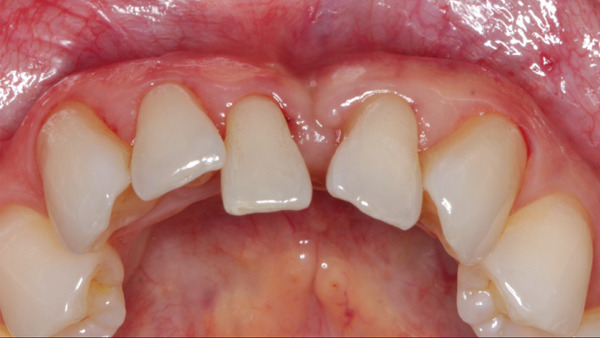
Presurgery, 45 days after tooth extraction. Occlusal view.

After the maintenance phase, tooth extraction was performed, and, after 45 days of healing [17], an early invagination formation could be evidenced, likewise interproximal and buccal recessions (Figures 4 and 5). Periodontal plastic surgery was performed.

**Figure 6:**
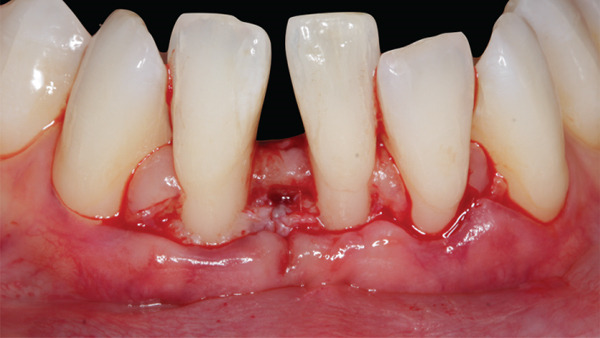
Flap design: envelope buccal flap with submarginal incisions toward the platform in the edentulous area.

The chosen surgical approach was a “connective tissue platform technique” for edentulous areas described by Zucchelli et al. [18]. Two parallel horizontal incisions (3 mm apart from each other) were placed on the occlusal surface of the edentulous area and buccally continued like a coronally advanced flap, following the principles established for the treatment of multiple gingival recessions in mucogingival surgery [19]. The flap itself was designed as an envelope and was extended from the distal surface of the left lateral incisor to the distal aspect of the right lateral incisor; oblique submarginal incisions were performed and directed toward the edentulous site. Flap elevation proceeded apicocoronally in a split‐full‐split manner (Figure 6).

**Figure 7:**
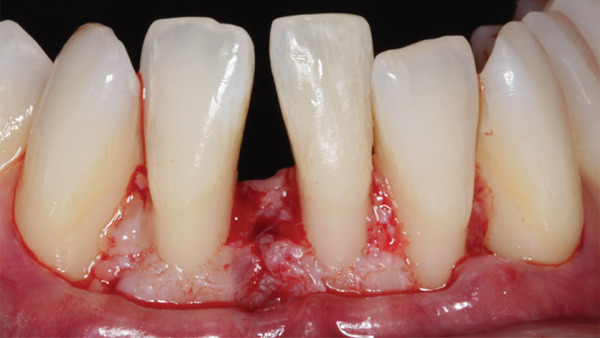
Envelope buccal flap elevation (split‐full‐split) and papillae de‐epithelialization.

Coronal advancement of the buccal flap was obtained by two split‐thickness incisions: one deep, releasing the muscle insertions from the periosteum, and one superficial, detaching the muscle inserting in the inner aspect of the flap′s lining mucosa. Afterward, de‐epithelialization of the anatomical papillae produced connective tissue beds into which the surgical papillae of the advanced flap would later be sutured. The occlusal surface of the edentulous area was also de‐epithelialized to create a full connective tissue platform acting as the recipient site for the connective tissue graft (CTG) (Figure 7).

**Figure 8:**
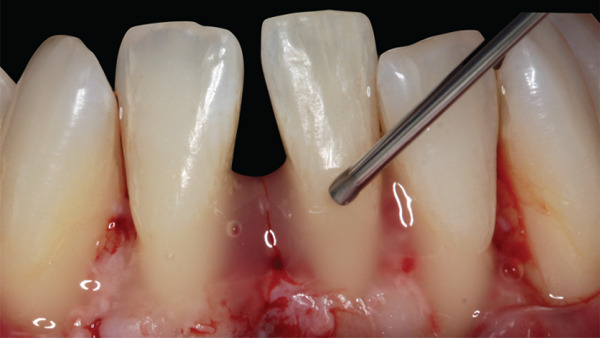
Root surface treatment (PrefGel and Emdogain).

Roots were treated mechanically and chemically [20], using curettes first and then 24% EDTA (PrefGel Straumann) for 2 min to remove smear layer, and after rinsing profusely, an enamel matrix derivative (Emdogain [EMD] 0.15 mL Straumann) gel was applied for 1 min [21, 22] (Figure 8).

**Figure 9:**
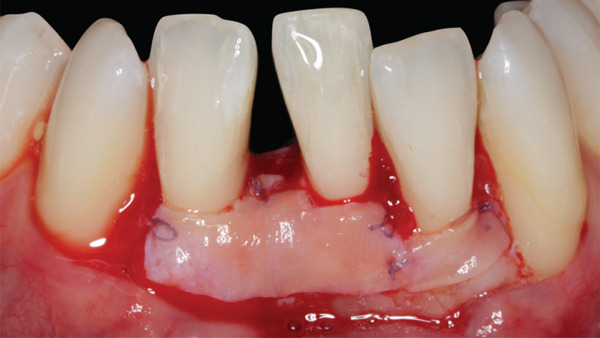
Suture of the de‐epithelialized free gingival graft harvested from the palate.

Two CTGs were harvested from the palate through a de‐epithelialized free gingival graft technique [23]. Both had a rectangular shape, but the larger one had a pedicle in the middle to treat the horizontal and vertical components of the soft tissue defect. The idea was to extend the grafts mesiodistally until the adjacent lateral incisors to improve their phenotype. Then, they were secured at the base of the anatomical papillae and anchored to the connective tissue of the platform with simple interrupted sutures (Figure 9).

**Figure 10:**
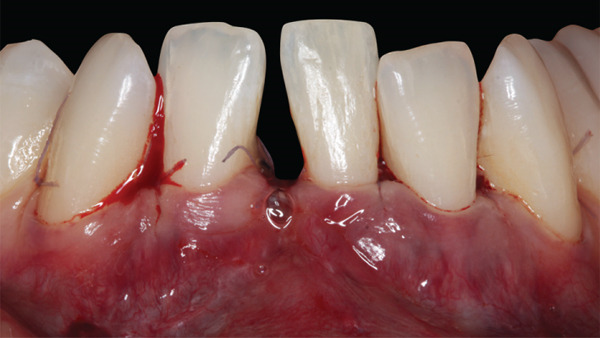
Final suture in a more coronal position.

Finally, the surgical papillae were anchored to their corresponding de‐epithelialized anatomical papillae using sling sutures suspended around the lingual cingulum of the treated teeth, which allowed the buccal flap to adapt closely to the dental crowns. A horizontal mattress suture, followed by single interrupted sutures, was performed to achieve complete closure of the horizontal incisions in the edentulous space and to facilitate primary intention wound healing over the CTGs used for soft tissue augmentation. All sutures used were 6‐0 PGA (ARYAN, Kalos) (Figure 10).

**Figure 11:**
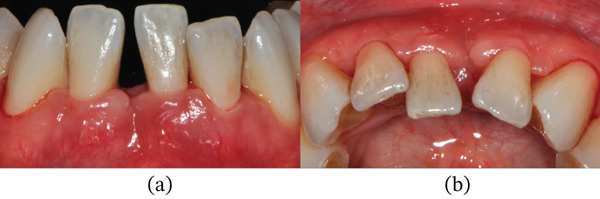
One‐month follow‐up: (a) buccal and (b) occlusal view.

Postoperative care instructions were given, healing was uneventful, and sutures were removed 15 days later. One‐month healing is shown in Figure 11.

## 3. Results

**Figure 12:**
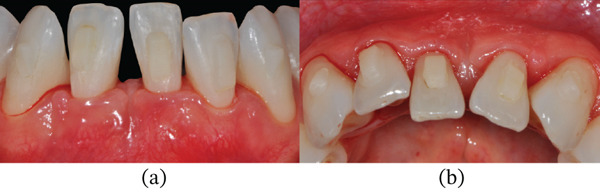
Four‐month follow‐up: (a) buccal and (b) occlusal view. Orthodontic treatment had begun.

Four months after healing, the orthodontic treatment was initiated with Invisalign aligner system (Figure 12). After 11 months of active treatment and 4 months of refinement, it was finished. Then, upper and lower thermoformed removable retainers were used.

**Figure 13:**
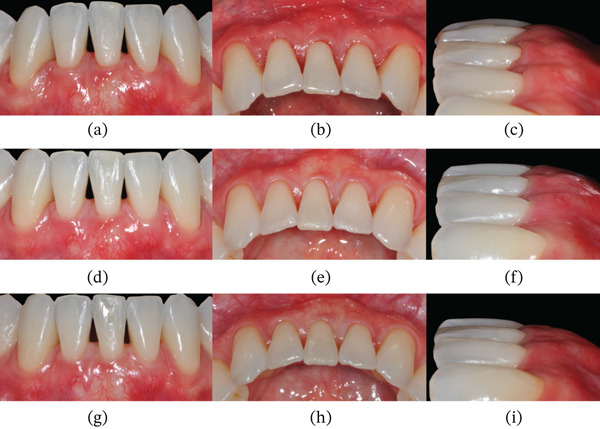
One‐year follow‐up: (a) buccal, (b) occlusal, and (c) lateral view. Two‐year follow‐up: (d) buccal, (e) occlusal, and (f) lateral view. Three‐year follow‐up: (g) buccal, (h) occlusal, and (i) lateral view.

**Figure 14:**
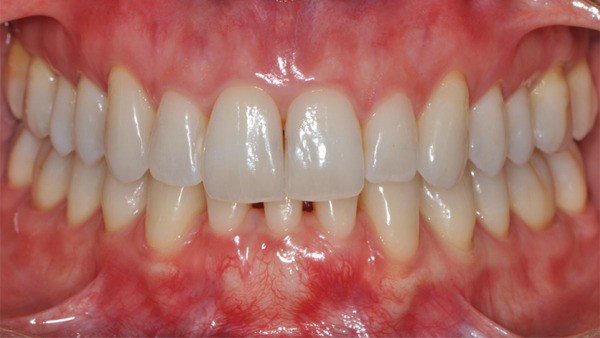
Three‐year follow‐up. Frontal view.

**Figure 15:**
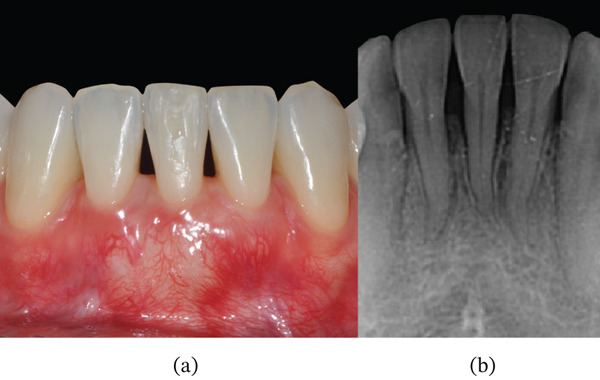
Three‐year follow‐up: (a) clinical and (b) radiographic view.

Follow‐up is shown at 1 (Figure 13), 2 (Figure 13), and 3 years (Figures 13, 14, and 15).

Three years after this multidisciplinary approach, soft tissue augmentation in vertical and horizontal dimensions was accomplished. After lower incisor extraction, a satisfactory orthodontic result was obtained. The overjet remained stable despite the Bolton discrepancy of the maxillary lateral incisors, and an adequate overbite was preserved. Anterior guidance was established, which permitted functional mandibular movements without posterior interferences and ensured proper root parallelism in the mandibular incisor region. Consequently, correct intercuspation in the posterior segment was maintained. A reasonable esthetic outcome was achieved. The soft tissue contour (scalloped outline), thickness, texture, color, and gingival margins of the anteroinferior area were well integrated with the adjacent gingival tissues, and, despite the absence of complete papilla height, there is no recession under clinical and visual evaluation (Figures 13, 14, and 15).

The patient reported high satisfaction with the treatment outcome at all follow‐up visits, with no subjective perception of recession at 3 years. This is consistent with recent evidence showing that patient‐reported esthetic satisfaction following root coverage procedures is consistently high and that even partial gingival margin improvement is sufficient to generate meaningful subjective benefit [24].

## 4. Discussion

The need for an interdisciplinary approach in orthodontic treatment, which often involves tooth extraction, is not new [25]. The extraction of a lower incisor constitutes a therapeutic alternative limited to certain occlusal situations [26], mainly those that involve Class I dental malocclusions with severe lower anterior crowding. It is also important to have the following specifications: normal dentition with perfect buccal interdigitation, Bolton discrepancy on lateral upper incisors, and severe lower anterior crowding between lower canines with a lack of space for almost one lower incisor [27, 28]. The case presented here met these conditions, and the right central incisor was the chosen one because of its malposition and gingival recession. Nevertheless, this approach frequently results in the invagination of the soft tissues or emergence of clefts [3–6] that should be solved to reduce or avoid the risk of orthodontic relapse and/or inadequate plaque control [2, 11]. In fact, several surgical treatments have been proposed to treat GCs, to prevent its formation, such as a socket preservation technique with a nonresorbable membrane after tooth extraction [29], or to treat them, with gingival excision [30, 31] or guided bone regeneration when alveolar bone is involved [32].

To the authors′ knowledge, the present case report is the first reported in the literature treating a GC associated with a mandible incisor extraction with a mucogingival approach using the “connective tissue platform technique” for edentulous areas described by Zucchelli et al. [18]. It has been demonstrated how this surgical approach was able to accomplish horizontal and vertical soft tissue augmentation in a single surgical step in the presence of a localized alveolar ridge defect, especially in the maxillary anterior dentition. In fact, our clinical results after 4 months showed a resolution of the mucogingival defect and its improvement over time through the 3‐year follow‐up. The envelope design allowed for an increased thickness and improved the phenotype of the adjacent teeth with CTGs, especially as they were going to receive facial movements, to prevent further recession formation [12, 33]. The selection of the graft harvesting technique was based on its histological characteristics; as it was closer to the epithelium, the graft was denser and more stable than a deeper one (more glandular and fattier) [23]. Furthermore, the use of EMD may have influenced the histological healing pattern through the induction of biological events associated with periodontal regeneration and soft tissue healing, given its properties of enhancing angiogenesis and local growth factor expression. However, its role in clinical benefits in the treatment of gingival recessions remains unclear [34].

Other published approaches include strategies for improving gingival and dentoalveolar conditions in patients undergoing orthodontic treatment. Among these, surgically facilitated orthodontic therapy (SFOT)—which encompasses corticotomy‐assisted orthodontics and periodontally accelerated osteogenic orthodontics (PAOO)—has been described as a proactive approach that augments dentoalveolar bone volume and expands the orthodontic boundary conditions prior to or concurrent with tooth movement [35].

The determination of the optimal timing for applying orthodontic forces (e.g., in the early healing phase or 6 months after surgery) needs to be further investigated with randomized comparative clinical studies [36]. The literature is controversial and reports results mainly after a guided bone regeneration approach. Therefore, this concern remains unclear, although there seems to be agreement that the longer the movement is delayed, the lesser the risk [37]. In the present case, the orthodontic treatment was initiated 4 months after surgery. At this time, it has been reported that, histologically, the epithelium had recovered its normal shape, thickness, and appearance, whereas the underlying connective tissue shows a well‐organized structure with dense collagen fibers and mature vessels [38].

As already widely reported in the literature, the role of periodontal supportive therapy with careful examination of periodontal tissues is of fundamental importance for successful long‐term results. In the present case, it was performed routinely every 6 months due to the patient′s low periodontal risk factors [39]. This, along with high patient compliance and absence of periodontal inflammation, allowed satisfactory long‐term results without causing irreversible damage to periodontal tissues [38].

## 5. Conclusions

Orthodontic closure of extraction sites frequently gives rise to gingival invagination, which to date remains a diagnostic challenge for clinicians. The present paper reports a perio‐ortho combined treatment in a patient with a gingival invagination after mandible incisor extraction and gingival recessions, which were treated with a periodontal plastic surgery approach prior to an orthodontic treatment.

A deep understanding of periodontology and orthodontics, combined with strong collaboration among professionals, expands the range of treatment choices in various situations. A multidisciplinary approach enhances the management of function and esthetics.

In the present clinical case, a 3‐year follow‐up success can be achieved after an additional application of a periodontal plastic surgery technique, which, combined with orthodontic treatment, solved a multidisciplinary treatment challenge.

The substantial absence of scientific evidence highlights the need for future research in certain directions, involving well‐designed studies aimed at delivering evidence‐based treatments to patients.

## Author Contributions

Conceptualization and methodology: M.R., J.A., and A.P.; formal analysis and investigation: M.R. and J.A.; data collection: J.A. and J.C.C.; data curation: M.R., J.A., and J.C.C.; writing—original draft preparation: M.R. and J.A.; writing—review and editing: M.R., J.A., and A.P.; supervision: A.P.; project administration: A.P.

## Funding

No funding was received for this manuscript.

## Disclosure

All authors have read and agreed to the published version of the manuscript. J.A. (corresponding author) had full access to all of the data in this study and takes complete responsibility for the integrity of the data and the accuracy of the data analysis.

## Ethics Statement

The study was conducted in accordance with the Declaration of Helsinki and approved by the Institutional Review Board (University of Buenos Aires; Res CD 983/17).

## Consent

This is a single‐anonymized case report for which informed consent was obtained.

## Conflicts of Interest

The authors declare no conflicts of interest.

## Data Availability

All data generated or analyzed during this study are included in this published article.
